# CircEPSTI1 Promotes the Proliferation of HER2-Positive Breast Cancer Cells via circEPSTI1/miR-145/ERBB3 Axis

**DOI:** 10.1155/2022/1028851

**Published:** 2022-08-26

**Authors:** Yue Zhang, Duxun Tan, Yi Xie, Linyu Wu, Song Wu, Yuhui Tang, Yongzhou Luo, Xiangsheng Xiao, Xing Li

**Affiliations:** ^1^Sun Yat-sen University Cancer Center, State Key Laboratory of Oncology in South China, Collaborative Innovation Center for Cancer Medicine, Guangzhou, Guangdong, China; ^2^Kangyuan Hospital, Guangzhou, Guangdong, China

## Abstract

Breast cancer is the most common type of cancer worldwide. There are great challenges in the prevention and treatment of breast cancer. In this study, we explored the molecular and biological mechanisms of circular RNA circEPSTI1 (has_circ_0000479) in the regulation of HER2-positive breast cancer cells. The expression of CircEPSTI1, microRNA miR-145, and ERBB3 in HER2-positive breast cancer cells was evaluated by qRT-PCR and western blot assays. Cell proliferation was assessed by CCK-8. Wound-healing and transwell migration assays were performed to evaluate cell migration. A transwell invasion assay was performed to detect cell invasion. The interaction of miR-145, circEPSTI1, and ERBB3 was confirmed bydual-luciferase reporter and RIP assays. CircEPSTI1 was upregulated in the HER2-positive breast cancer tissues and cells. Knockdown of circEPSTI1 inhibited SKBR3 and BT474 cell proliferation, migration, and invasion. Mechanistically, circEPSTI1 directly targeted miR-145, and miR-145 was a downstream mediator of circEPSTI1 in modulating the proliferation, migration, and invasion of SKBR3 and BT474 cells. ERBB3 was identified as a direct and functional target of miR-145 in HER2-positive breast cancer cells. Our findings demonstrate that circEPSTI1, an overexpressed circRNA in HER2-positive breast cancer, promotes the proliferation, migration, and invasion of SKBR3 and BT474 cells through the miR-145/ERBB3 axis.

## 1. Introduction

Breast cancer is one of the most common cancers worldwide, accounting for about 30% of female cancers, and has a mortality-to-incidence ratio of 15% [1]. Globally, the incidence of breast cancer ranges between 27 in 100,000 (for Africa and East Asia) and 97 in 100,000 (for North America) [2]. Human epidermal growth factor receptor 2 (HER2/ERBB2) positive breast cancer accounts for 20–25% of all breast cancers. Although the development of HER2-directed therapy has revolutionized the mode and outcome of treatment in patients, the high toll of HER2-positive breast cancer deaths persists [3, 4]. Currently, the development of drug resistance, unfavorable side effects of TKIs, and tumor metastasis are big challenges in the management of breast cancer [3].

CircRNA is a type of noncoding RNA that forms a covalently closed continuous loop [5]. Based on this feature, circRNAs exhibit a variety of functions, and many of them were discovered in recent years [6]. CircRNAs modulate target gene expression by binding to RNA-binding proteins [7]. In addition, it has been shown that circRNA can sponge miRNAs to control the function of miRNAs [8]. A number of studies have found that altered circRNA expression in various human cancers and circRNA play crucial roles in tumorigenesis.

MicroRNAs (miRNAs) also belong to noncoding RNA that play a central role in cell proliferation, differentiation, and survival by targeting complementary mRNAs, contributing to mRNA translational inhibition or degradation [9]. Wei et al. [10] found that miR-422a binds to the 3′-UTR of MAPK1 and activates Raf/MEK/ERK signaling pathway to promote colorectal cancer cell proliferation. Ferlay et al. confirmed that miR-21 affects the p53 signaling pathway [11], a well-known tumor suppressive gene [12, 13]. Besides, as miRNAs are associated with cancer metastasis, a new clinical diagnostic target based on miRNA signatures is being studied to identify subtypes of breast cancer and predict metastasis or treatment resistance [14–16]. miRNAs are capable of acting as biomarkers due to their stability and the ability to resist repeated freezing and thawing [17].

ERBB3 is one of the ErbB family and plays a vital role in the development and progression of tumors [18]. Because of the success of targeting EGFR and ErbB2, targeting ERBB3 is becoming increasingly interesting. A similar strategy can be used to target the latter, as all family members have great similarities in structure. ERBB3 plays an important role in several compensatory processes of tumorigenesis and the emergence of traditional cancer drugs. At the moment, there are no approved anti-ERBB3 therapies in clinical practice, whereas many drugs are at different stages of clinical development [18].

Here, we found that circEPSTI1 was upregulated in 20 paired HER2-positive breast cancer specimens compared to their adjacent normal tissues. Based on the significant differential expression of circEPSTI1, we were supposed to explore its role in HER2-positive breast cancer. Knockdown of circEPSTI1 observably inhibited cell proliferation, migration, and invasion. Furthermore, circEPSTI1 could bind to miR-145 and regulate the expression of ERBB3. Thus, circEPSTI1 could be used as a novel biomarker and potential therapeutic target for breast cancer.

## 2. Materials and Methods

### 2.1. Ethical Standards

This study was approved by the Ethics Committee of Sun Yat-Sen University Cancer Center Health Authority (GZR2017-163). All the procedures were performed strictly in accordance with the ethical standards of the Declaration of Helsinki (as revised in 2013). Patient consent was obtained before the study commenced.

### 2.2. Cell Culture and Transfection

SKBR3 and BT474 cells were obtained from the American Type Culture Collection (ATCC, VA, USA). Cells were cultured in DMEM media containing 10% FBS (Gibco, USA, Cat.# 16140071) and 1% penicillin-streptomycin (Gibco) at 37°C in a 5% CO_2_ incubator. After resuscitation from frozen aliquots, all cells were passaged in the laboratory for less than 6 months and were verified by DNA fingerprinting every 6 months. All si-RNAs, plasmids, and viruses used in this study were obtained from GenePharma (Shanghai, China). Lipofectamine 3000 (Invitrogen, Carlsbad, CA, USA) was used for cell transfection. 48 hours and 72 hours after the transfection, cells were collected for qRT-PCR and western blotting study.

### 2.3. qRT-PCR Experiment

Total RNA was extracted using TRIzol reagents (Invitrogen, USA). The PARISTM Kit (Invitrogen) was used to isolate cytoplasmic and nuclear RNA. cDNA was synthesized by the PrimeScript RT reagent kit (Takara, Dalian, China) or mirVanaTM qRT-PCR miRNA Detection Kit (Ambion, Austin, TX, USA). The U6 or GAPDH was used as the housekeeping gene to control and evaluate the abundances of target transcripts. The relative expression was assessed by the 2^−ΔΔCT^ method.

### 2.4. RNase R Digestion Experiment

RNA isolated from SKBR3 and BT474 cells was divided into two groups: treatment with RNase *R* (Epicentre Technologies, Madison, USA, Cat.# RNR07250) and buffer control. 2 *μ*g of total RNA was mixed with RNase *R* (3 U/*μ*g) in the RNase *R* group for 20 mins at 37°C. *β*-Actin was used as an internal control.

### 2.5. Cell Proliferation Assay

Cell Counting Kit-8 (CCK-8) (GLPBIO, USA) was used to evaluate cell proliferation. In 96-well plates, 1 × 10^3^ per well of SKBR3 or BT474 cells were seeded. After 24, 48, 72 and 96 hours, each well was added with 10 *μ*l CCK-8 mixture mixed with 100 *μ*l medium. After 2 h of incubation at 37 °C, absorbance was measured at 450 nm in a microtiter plate reader (BioTek EPOCH2, Winooski, VT).

### 2.6. Transwell Migration and Invasion Assay

During the migration assay, we seeded transfected SKBR3 or BT474 cells in the top chamber with a no-serum medium and added a medium with 20% FBS into the lower chamber. During the invasion assay, we covered the transwell inserts (Fisher Scientific, MA, USA) with Matrigel (BD, Franklin Lakes, USA), then seeded transfected SKBR3 or BT474 cells in the top chamber with no-serum medium, and added medium with 20% FBS into the lower chamber. 24 hours after incubation, cells on the upper surface of the transwell chamber were gently removed with a cotton swab, and cells on the lower surface were fixed with methanol. Then the cells were stained with 0.5% crystal violet (Solarbio), imaged, and counted.

### 2.7. Wound-Healing Assay

In 6-well cell culture dishes, 1 × 10^5^ transfected SKBR3 cells were seeded in each well. When the cells achieved about 95% confluence, a sterile 200-*μ*L pipette tip was used to create a scratch across the cell monolayer. Images were photographed at 0 and 24 h. Wound closure analysis was performed using ImageJ software (National Institute of Health, Bethesda, MD, USA).

### 2.8. Luciferase Reporter Assay

By cloning fragments of the circEPSTI1 or ERBB3 3′ UTR that contain the predicted wild-type or mutant binding sequences of miR-145 into the pmirGLO vector (Promega Corporation, Fitchburg, USA), we were able to detect both wild-type and mutant binding sequences of miR-145. Luciferase reporter vectors circEPSTI1 WT, circEPSTI1 MUT, ERBB3 3′ UTR WT, and ERBB3 3′ UTR MUT were formed. SKBR3 or BT474 cells were transfected with the indicated vector and miR-ctr or miR-145. Using the Dual-Luciferase Reporter Assay Kit (Promega), the luciferase activity was examined 48 hours later.

### 2.9. RNA Immunoprecipitation Assay

SKBR3 or BT474 cells were planted in 6-well plates and co-transfected when proliferation was 60%–70% with strict requirements. After 48 hours, the Magna RIP Kit (Millipore) was used to perform the RIP assay. An RNA immunoprecipitation assay was performed with an anti-Ago2 antibody (Millipore) for the RIP assay on Ago2.

### 2.10. Western Blotting

Protein was isolated with RIPA lysis buffer (Solarbio, #R0020-RIPA) with a proteinase inhibitor (PMSF, 100 mM, Biosharp) and strictly quantified with a BCA kit (Thermo Fisher Scientific Inc., Rockford, IL). The extracted protein was electrophoresed on SDS-polyacrylamide gels, and the resulting gel was electroblotted onto Clear Blot membrane-p (ATTO, Tokyo, Japan). The polyvinylidene difluoride membranes were blocked with 5% fat-free milk for 1 hour at room temperature and then incubated with primary antibodies overnight at 4 °C. The western blot analysis was performed with the following antibodies: anti-ERBB3 (1 : 1000 dilution, Proteintech Group Inc., Chicago, IL) and *β*-tubulin (1 : 5000 dilution, Cell Signaling Technology, MA). The membranes were further incubated in a goat anti-rabbit IgG H&L (1 : 5000 dilution, Abcam, Cambridge, USA) and ECL reagents (Solarbio, China,#SW2040) were used to detect the protein.

### 2.11. Statistical Analysis

SPSS 25.0 software was used for statistical analysis. The *t*-tests and *χ*^2^ tests were performed for comparisons between groups. Unless otherwise noted, the mean ± SEM of triplicate independent experiments are used to present data. *P* < 0.05 was considered statistically significant.

## 3. Results

### 3.1. CircEPSTI1 Is Increased in HER2-Positive Breast Cancer Cells and Tissues

**Figure 1:**
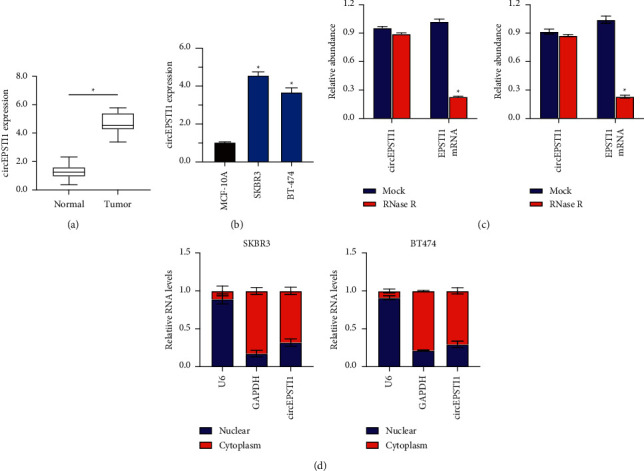
CircEPSTI1 (circ0000479) is increased in HER2-positive breast cancer cell lines and tissues. (a) Expression of circEPSTI1 in HER2-positive breast cancer tissues and matched normal tissues. (b) Expression of circEPSTI1 in normal and HER2-positive breast cancer cell lines. (c) RNase R digestion confirmed the circular structure of circEPSTI1 in SKBR3 (left) and BT474 cells (right). (d) The qRT‐PCR evaluated the levels of U6, GAPDH, and circEPSTI1 in nuclear and cytoplasmic fractions. ^*∗*^*P* < 0.05.

The first step was to conduct qRT-PCR in order to characterize the level of circEPSTI1 in HER2-positive breast cancer tissues. The result demonstrated that HER2-positive breast cancer tissues had a significantly higher circEPSTI1 expression compared with nearby normal tissues (Figure 1(a)). In addition, circEPSTI1 expression was detected to be increased in SKBR3 and BT474 cells compared with MCF-10A cells (Figure 1(b)). CircRNAs showed an apparent resistance to the degradation mediated by exonuclease. RNase *R* was employed to treat the total RNA from the HER2-positive breast cancer cell lines. The result confirmed the circular structure of circEPSTI1 by RNase *R* digestion experiments in SKBR3 and BT474 cells (Figure 1(c)). The results of the subcellular fraction assay showed that circEPSTI1 was mostly enriched in the cytoplasm of SKBR3 and BT474 cells (Figure 1(d)).

### 3.2. CircEPSTI1 Promotes the Proliferation, Migration, and Invasion of HER2-Positive Breast Cancer Cells

**Figure 2:**
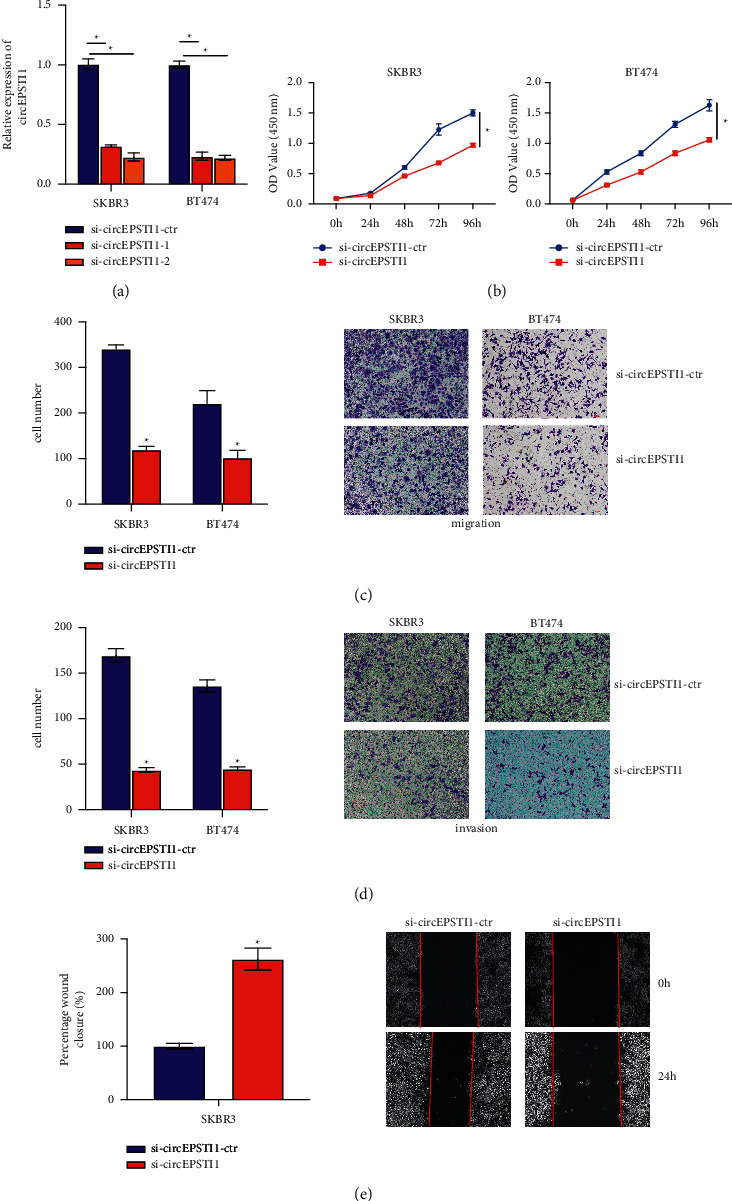
Knockdown of circEPSTI1 suppresses HER2-positive breast cancer growth. (a) Si-circEPSTI1 knocked down circEPSTI1 successfully in SKBR3 and BT474 cells. (b) Cell Counting Kit-8 assay evaluated cell proliferation in SKBR3 and BT474 cells. (c) Transwell migration assay evaluated the migration of SKBR3 and BT474 cells. (d) Transwell invasion assay evaluated migration of SKBR3 and BT474 cells. (e) The migration of SKBR3 cells was assessed by a wound-healing assay. ^*∗*^*P* < 0.05.

Based on the upregulation of circEPSTI1 in HER2-positive breast cancer, si-circEPSTI1 was transfected to knock down circEPSTI1 to explore its biological function in HER2-positive breast cancer SKBR3 and BT474 cells. The transfection was successfully performed (Figure 2(a)). The CCK-8 assay indicated that circEPSTI1 downregulation significantly weakened the proliferation of the cells (Figure 2(b)). The Transwell migration and invasion assay confirmed that circEPSTI1 knockdown strongly hindered the migration and invasion processes of these cells (Figures 2(c) and 2(d)). Also, the wound-healing assay confirmed that knockdown of circEPSTI1 obviously suppresses the migration of SKBR3 cells (Figure 2(e)). These results indicated that circEPSTI1 knockdown suppressed the proliferation, migration, and invasion of HER2-positive breast cancer cells.

### 3.3. CircEPSTI1 Targets miR-145 to Facilitate HER2-Positive Breast Cancer Progression

**Figure 3:**
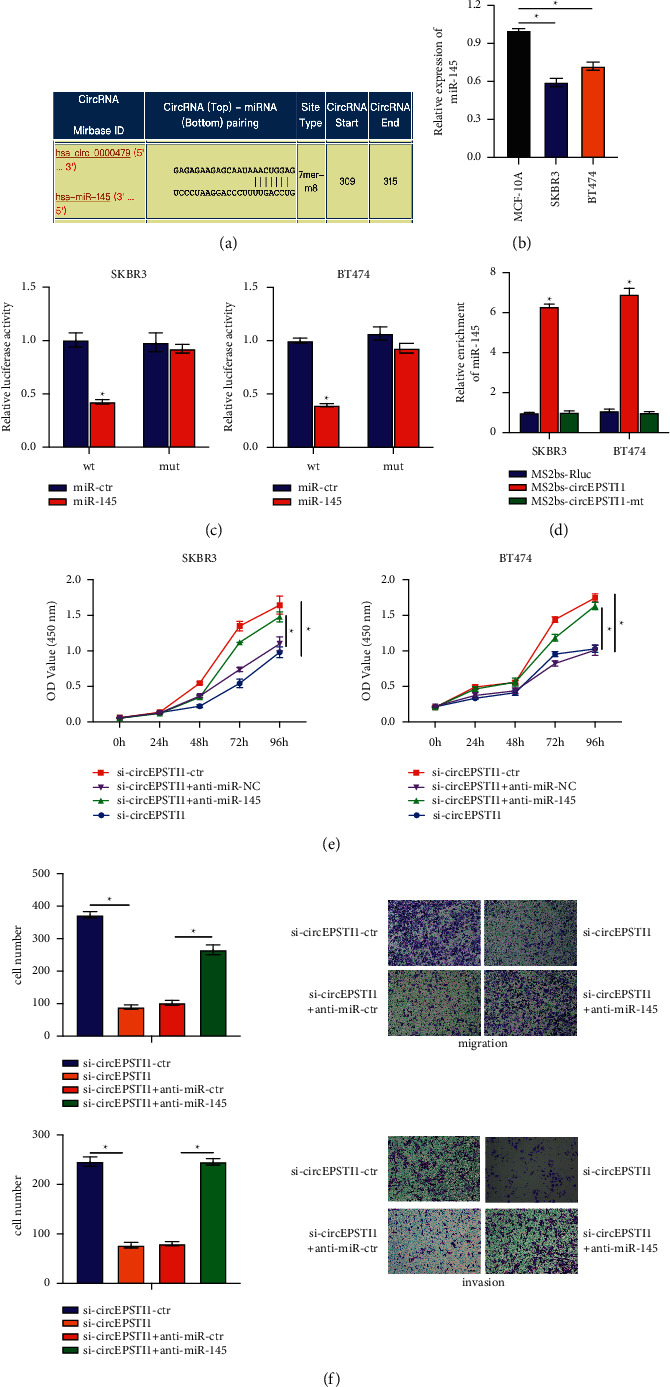
circEPSTI1 functions as a sponge for miR-145 in SKBR3 and BT474 cells. (a) Predicted binding sites of miR-145 within the circEPSTI1 sequence. (b) Expression of miR-6085 in SKBR3 and BT474 cells. (c) Relative luciferase activity of SKBR3 and BT474 cells co-transfected as required. (d) MS2-based RIP assay in SKBR3 and BT474 cells transfected with MS2bs-circEPSTI1, MS2bs-circEPSTI1-mt, or MS2bs-Rluc. (e) CCK-8 assay evaluated cell proliferation in SKBR3 and BT474 cells transfected separately with si-ctr, si-circESPTI1, si-circESPTI1 + anti-miR-ctr, or si-circESPTI1 + anti-miR-145. (f) Transwell migration and invasion assays evaluated migration and invasion ability of SKBR3 cells transfected separately with si-ctr, si-circESPTI1, si-circESPTI1 +  anti-miR-ctr, or si-circESPTI1 +  anti-miR-145. ^*∗*^*P* < 0.05.

As we had found, circEPSTI1 was mainly distributed in the cytoplasm (Figure 1(d)), suggesting that circEPSTI1 might function as a miRNA sponge to adsorb miRNAs. Hence, we investigated the possible interactions between circRNA and miRNA in the Circular RNA Interaction Group (https://circinteractome.nia.nih.gov/index.html) and located complementary sites for miR-145 in the circEPSTI1 sequence (Figure 3(a)). In addition, the expression of miR-145 was obviously decreased in SKBR3 and BT474 cells (Figure 3(b)). Then a luciferase assay was performed to explore whether miR-145 could bind to circEPSTI1. Luciferase intensity receded when co-transfected with the luciferase reporter gene and miR-145 mimic (Figure 3(c)). To further confirm the direct binding of circEPSTI1 and miR-145, we performed an RIP assay based on MS2bp-MS2bs. The RIP assay showed that miR-145 was mostly enriched in the MS2bs-circEPSTI1 group, indicating a specific interaction between circEPSTI1 and miR-145 (Figure 3(d)). According to the above results, we further explored whether circESPTI1 modulated the proliferation, migration, and invasion of HER2-positive breast cancer cells by directly targeting miR-145. SKBR3 and BT474 cells were divided into 4 groups: si-ctr, si-circESPTI1, si-circESPTI1 + anti-miR-ctr and si-circESPTI1 + anti-miR-145. By cell proliferation experiment (Figure 3(e)), the proliferative capacity, weakened by circESPTI1 knockdown, could be restored by co-transfected miR-145 inhibitor. Transwell experiments showed that the migration and invasion ability, suppressed by circESPTI1 knockdown, was restored by co-transfected miR-145 inhibitor in SKBR3 cells (Figure 3(f)). These results suggest that circESPTI1 regulates the proliferation, migration, and invasion of HER2-positive breast cancer cells as a miR-145 sponge, promoting breast cancer progression. Overall, we confirmed that circEPSTI1 could interact with miR-145 and act as a decoy for miR-145.

### 3.4. CircEPSTI1 Regulates ERBB3 as miR-145 Sponge in HER2-Positive Breast Cancer Cells

**Figure 4:**
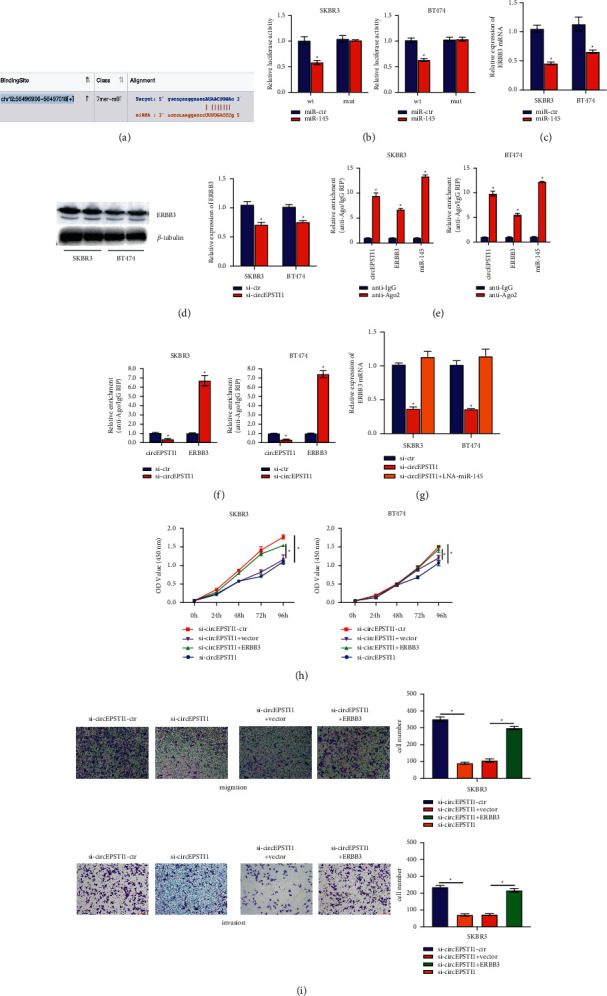
CircEPSTI1 regulates ERBB3 as mir-145 sponge in HER2-positive breast cancer cells. (a) Predicted direct binding sites of hsa-miR-145 within ERBB3 sequences. (b) Luciferase reporter assay was performed and the luciferase activity of cells co‐transfected with miR-145 mimics and luciferase reporter containing EPSTI1 3′UTR (wt) or mutant construct (mut) was tested. (c) The qRT‐PCR was used to determine the expression of ERBB3 in SKBR3 and BT474 cells transfected as described. (d) The expression of ERBB3 determined by western blot (left) and quantified (right). (e) RIP assay measured the enrichment of circEPSTI1, EPSTI1, and miR-145 on Ago2 relative to IgG. (f) RIP assay on Ago2 performed in SKBR3 and BT474 cells transfected as described. (g) qRT‐PCR performed to detect expression of ERBB3 in SKBR3 and BT474 cells transfected. (h) CCK-8 assay evaluated cell proliferation in SKBR3 and BT474 cells transfected. (i) Transwell migration and invasion assays evaluated migration and invasion ability of SKBR3 cells transfected. ^*∗*^*P* < 0.05.

To explore whether circEPSTI1 decoyed miR-145 to influence the expression of target genes, ENCORI (https://starbase.sysu.edu.cn/) was used to predict the target genes of miR-145 and ERBB3 was identified (Figure 4(a)). Luciferase activity markedly decreased with co-transfected miR-145 mimics by luciferase reporter assay (Figure 4(b)). Furthermore, we found that the expression of ERBB3 was inhibited by miR-145 (Figures 4(c) and 4(d)), suggesting that ERBB3 was a direct target of miR-145. CircEPSTI1, ERBB3, and miR-145 were mainly enriched to Ago2 by RIP assay on Ago2 (Figure 4(e)), suggesting that circEPSTI1 and ERBB3 are recruited to an Ago2-related RISC where they interact with miR-145. Additionally, knockdown of circEPSTI1 significantly decreased the enrichment of Ago2 to circEPSTI1 while increasing the enrichment of Ago2 to ERBB3 (Figure 4(f)), confirming that circEPSTI1 could bind to miRNAs and compete with ERBB3 as a ceRNA. Then, we investigated the expression of ERBB3 after knocking down circEPSTI1 and found it was inhibited, yet inhibiting miR-145 could reverse the repression (Figure 4(g)). These findings indicate that circEPSTI1 could act as a miR-145 sponge to ERBB3 expression via the ceRNA mechanism.

To verify whether miR-145 directly acted on ERBB3 to affect HER2-positive breast cancer cells, we divided SKBR3 and BT474 cells into 4 groups: si-ctr, si-circESPTI1, si-circESPTI1 + ERBB3-ctr, and si-circESPTI1 + ERBB3. CCK-8 assay was performed and showed that the suppressing proliferation caused by knocking down circEPSTI1 was basically recovered after co-transfection of ERBB3, contracting to si-circESPTI1 transfected in SKBR3 and BT474 cells (Figure 4(h)). Furthermore, transwell experiments showed that the weakening migration and invasion ability of cells caused by knocking down circEPSTI1 could be recovered after co-transfection of ERBB3 in SKBR3 cells (Figure 4(i)). These results indicated that miR-145 decreased ERBB3 transcription and translation, and circEPSTI1 promoted her2-positive breast cancer progression by regulating ERBB3 as a miR-145 sponge.

## 4. Discussion

Studies on CircRNAs have brought about many interesting findings, indicating that circRNAs play very important roles in the occurrence and development of various tumors [9, 19]. CircRNA is a type of noncoding RNA, although recent studies have shown that circRNA also has the potential to code for proteins [20]. Due to their special molecular structure, circRNAs exhibit outstanding stability in cells [21], and they are selective and specific in tissue and organ expression [22]. It has been suggested that circRNA expression is related to the developmental stage or aging [23]. Qiu et al. [24] found that circ103809 can directly modulate the function of miR-532-3p, block the G2/M phase of the cell cycle, and interfere with the EMT signaling cascade. Chen et al. [25] studied the expression profile of human circRNA in TNBC tissue and detected that circEPSTI1 (hsa_ circRNA_0000479) is a significantly upregulated circRNA. It significantly reduces the accumulation of various chemotherapeutic drugs in monastol-resistant cell lines [26]. In addition, circRNA may be involved in modulating immune responses. Studies have shown that circCDR1 plays a key role in immune and stromal cell invasion of breast cancer, especially CD8+ T cells, activating immune-related cells such as NK cells, M2 macrophages, and endothelial cells [27]. According to numerous studies, circRNAs can be used as a cancer marker for the diagnosis and prognosis of tumors.

Abundant studies have explored the function of miR-145 and found that miR-145 held many functions and had different effects on tumor. miR‐145 overexpression increased chemotherapeutic efficiency by inducing intracellular doxorubicin accumulation by suppressing MRP1 in breast cancer [28]. Sheng et al. [29]found that miR-145 had an inhibitory effect on colorectal cancer, reducing cell migration and invasion via suppressing P21-activated kinase 4 (PAK4). miR-145 could interact with circITCH to regulate its target gene RASA, promoting the progression of ovarian cancer [30].miR‐145 regulates the SOX2-Wnt/*β*-catenin axis to enhance chemosensitivity to demethoxycurcumin in gliomas [31]. Kou et al. [32] found that miR-145 inhibited tumor invasion via targeting PAK1 in bladder cancer cells and enhanced EMT through phosphorylating Snail. These findings indicate that miR-145 has a strong effect on tumor progression.

Upregulated ErbB3 plays a vital role in primary and acquired resistance against ErbB2 drugs, chemotherapy drugs, tyrosine kinase inhibitors (TKIs), and hormone therapy. Numerous studies have found that ErbB3 is overexpressed in breast, ovarian, prostate, colon, pancreatic, gastric, urinary, oral, and lung cancers [33]. In patients diagnosed with estrogen receptor (ER)-positive breast cancer, erbB3 upregulation occurs after treatment with fulvestrant, activating the MAPK/ERK pathway and leading to resistance to antiestrogen therapy [34]. In ERbB2 and ER-positive breast cancer patients, ErbB3 overexpression by ErbB2 manufacturing heterodimers, and activating the PI3K/AKT3 signaling pathway leads to tamoxifen resistance, thereby restraining forkhead box O3 (FOXO3a) transcription factors. Eventually, the nuclear estrogen receptor ER*α* is downregulated [35].

In HER2-positive breast cancer, we focused on circEPSTI1, revealed the role of miR-145 as its downstream molecule, and verified that ERBB3 is regulated by miR-145 and has significant changes in mRNA and protein levels, which provides a basis for understanding the mechanism of the occurrence and development of HER2-positive breast cancer, and provides the therapeutic possibility of tumor drug resistance, recurrence, and metastasis.

There were some limitations to this study. We only demonstrated that circEPSTI1 is a miRNA sponge. A large number of studies have confirmed that circRNAs have other roles such as communication with RNA-binding proteins (RBPs) [34], translation of proteins or peptides [36], and transcription regulation [37] that eventually produce a variety of different biological effects involved in the regulation of tumor cells, tumor microenvironment, angiogenesis, tumor immunity, and more [38]. In this study, we have elucidated the ability of circRNA as a miRNA sponge to promote the proliferation, migration, and invasion of HER2-positive breast cancer cells via targeting ERBB3. We have found one of the mechanisms of malignant behavior of HER2-positive breast cancer. However, the process involving circRNAs is still unclear. At present, there are a considerable number of ERBB3 targeted drugs in clinical and preclinical areas, such as Patritumab, Elgemtumab, Seribantumab, REGN1400, GSK2849330, Anti-NRG antibodies, and others [33]. Combined with their pharmacological mechanisms, it is of great significance to explore the potential role of circRNAs in guiding the treatment of HER2-positive breast cancer, reversing drug resistance, and improving prognosis. In addition, in biological basic research, it is best to combine experiments in vivo and related breast cancer clinical data to further illustrate the role and effect of circEPSTI1 in the body environment, which is conducive to the construction of a possible network of circRNAs as a biomarker in breast cancer occurrence, development, prognosis, medication guidance and other aspects of an application.

## 5. Conclusions

In this study, we elucidated that circEPSTI1 acts on ERBB3 by adsorbing miR-145, thereby promoting the proliferation, migration, and invasion of HER2-positive breast cancer cells. More importantly, since ERBB3 belongs to the ErbB family, there have been a large number of studies to explore the treatment mechanism and efficacy of ERBB3 targeting HER2-positive breast cancer, and our study complements the mechanism of the occurrence and development of HER2-positive breast cancer and provides possible effective markers and treatment ideas.

## Data Availability

The data are available from the corresponding author Xing Li upon request via email (lixing@sysucc.org.cn).

## Supplementary Materials

Supplementary Materials The primer sequences for qRT-PCR and sirna sequences used in this study are provided in supplementary file.
