# Supplementary material for: CircEPSTI1 Promotes the Proliferation of HER2-Positive Breast Cancer Cells via circEPSTI1/miR-145/ERBB3 Axis
Source: J Oncol. 2022 Aug 26;2022:1028851. doi: 10.1155/2022/1028851 (PMC9439903; doi:10.1155/2022/1028851)
Supplement: Supplementary Materials — The primer sequences for qRT-PCR and sirna sequences used in this study are provided in supplementary file. [file 1028851.f1.pdf]

The primer sequences for qRT-PCRs used in this study

Construct	Species	Direction	Sequence (5' - 3')
circEPSTI1	Human	Forward	AAGCTGAAGAAGCTGAACTC
		Reverse	GTGTATGCACTTGTGTATTGC
U6	Human	Forward	CTCGCTTCGGCAGCACA
		Reverse	AACGCTTCACGAATTTGCGT
β-actin	Human	Forward	AGCGAGCATCCCCCAAAGTT
		Reverse	GGGCACGAAGGCTCATCATT
GAPDH	Human	Forward	GGAGCGAGATCCCTCCAAAAT
		Reverse	GGCTGTTGTCATACTTCTCATGG
ERBB3	Human	Forward	GACAGAAGTGCCTGTCAAAGTG
		Reverse	GCCGTTTCAGTTCCAGTAATTC
miR-145	Human	Forward	GCCGCGTCCAGTTTTCCCAGG
		Reverse	GTGCAGGGTCCGAGGT

The sirna sequences used in this study

siRNA	Species	Sequences
si-NC	Human	UUCUCCGAACGUGUCACGUTT
si-circEPSTI1-1	Human	GCAAUACACAAGUGCAUACTT
si-circEPSTI1-2	Human	AGCAAUACACAAGUGCAUATT
